# Reducing Tolerance for SABA and OCS towards the Extreme Ends of Asthma Severity

**DOI:** 10.3390/jpm12030504

**Published:** 2022-03-21

**Authors:** Petros Bakakos, Konstantinos Kostikas, Stelios Loukides, Michael Makris, Nikolaos G. Papadopoulos, Paschalis Steiropoulos, Stavros Tryfon, Eleftherios Zervas

**Affiliations:** 1 1st University Department of Respiratory Medicine, National and Kapodistrian University of Athens, 157 72 Athens, Greece; petros44@hotmail.com; 2 Respiratory Medicine Department, School of Medicine, University of Ioannina, 451 10 Ioannina, Greece; ktkostikas@uoi.gr; 3 2nd University Department of Respiratory Medicine, National and Kapodistrian University of Athens, 157 72 Athens, Greece; loukstel@med.uoa.gr; 4 Allergy Unit, 2nd Department of Dermatology and Venereology, National and Kapodistrian University of Athens, 157 72 Athens, Greece; mmakris.attikon@gmail.com; 5 Allergy Department, 2nd Pediatric Clinic, National and Kapodistrian University of Athens, 157 72 Athens, Greece; ngpallergy@gmail.com; 6 Department of Respiratory Medicine, Medical School, University General Hospital, Democritus University of Thrace, 691 00 Alexandroupolis, Greece; steiropoulos@yahoo.com; 7 Respiratory Department of NHS, “G. Papanikolaou” General Hospital, 570 10 Thessaloniki, Greece; stavrostryfon@yahoo.gr; 8 7th Respiratory Department, Athens Chest Hospital “Sotiria”, 115 27 Athens, Greece

**Keywords:** asthma, mild asthma, severe asthma, short-acting beta-agonists, oral corticosteroids

## Abstract

Asthma is a heterogeneous chronic inflammatory airway disease that imposes a great burden on public health worldwide. In the past two years, fundamental changes have been addressed in the Global Initiative for Asthma (GINA) recommendations focusing mainly on the management of mild and severe asthma. The use of as-needed treatment containing inhaled corticosteroids plus fast-acting bronchodilators (either short or long-acting formoterol) in mild asthma has dominated the field, and both randomized and real-world studies favor such an approach and associate it with fewer exacerbations and good asthma control. At the same time, the effort to diminish the use of oral steroids (OCS) as maintenance treatment in severe asthma was substantially accomplished with the initiation of treatment with biologics. Still, these options are available at the moment only for severe asthmatics with a T2-high endotype, and relevant studies on biologics have yielded, as a primary outcome, the reduction or even cessation of OCS. Accordingly, OCS should be considered as a temporary option, mainly for the treatment of asthma exacerbations, and as a maintenance treatment only for a minority of patients with severe asthma, after ensuring good inhaler technique, modification of all possible contributory factors and comorbidities, and optimized pharmacotherapy using all other add-on treatments including biologics in the armamentarium of anti-asthma medication.

## 1. Introduction

Asthma is a chronic inflammatory airway disease characterized by heterogeneity. This heterogeneity is mainly driven by inflammatory changes, therapeutic interventions, and other contributable factors such as age of onset, gender, and atopy [1].

Therapeutic interventions have been extensively evaluated in the last decade. The main changes were the initiation of treatment with biologics, the effort to diminish the use of oral steroids as maintenance treatment in the severe forms of the disease, and finally, the use of as-needed treatment containing inhaled steroids plus bronchodilators, either short or long-acting [2,3,4].

Treatment with oral steroids (OCS) as maintenance treatment was the option of choice for patients with severe asthma who remained uncontrolled despite the use of high doses of (inhaled corticosteroids) ICS [5]; however, the above intervention was clearly characterized by the presence of long-term side effects [6]. The OCS sparing effect was considered as the main outcome of different studies with different agents, with biologics to be considered as the most effective option in that process [7]. Furthermore, a strategy of OCS sparing was recently published focusing on either methodology or/and safety [8].

The as-needed approach as a maintenance or/and reliever option for patients with asthma is currently a part of the GINA guidelines. The latest version of GINA clearly distinguishes a preferential approach with the use of a regime containing ICS plus formoterol from an alternative approach following the old traditional way of treatment with short-acting beta-agonists (SABA). This symptom-based approach highly depends on patients recognizing symptoms and mainly targets the overuse of SABAs as well as the rate of exacerbations in patients with mild asthma. Pragmatic and randomized trials containing different regimes have already confirmed the beneficial effect of this treatment option in patients with mild asthma [9,10,11].

In the current review, we discuss the two aforementioned treatment-based approaches as part of our treatment strategy in different forms of the disease. We summarize the currently available data, and we aim to determine the strengths and the limitations that potentially drive the above treatment options. Finally, we focus on some future research gaps that need to be addressed to eliminate any currently existing limitations.

## 2. Reducing Inappropriate Use of SABA in Mild Asthma

### 2.1. The Use of SABA in Asthma—The Story so Far

Despite the observed significant reductions in asthma mortality and hospitalization rates and significant advances in pharmacotherapy in recent years, asthma remains uncontrolled in a significant proportion of patients worldwide [12,13].

Patients with symptomatic uncontrolled asthma tend to increase their reliever medication, often a short-acting β_2_-agonist (SABA), rather than re-adjusting their controller medication as proposed in written asthma plans. Conceptually, this is in line with the somehow paradoxical concept that in almost all guideline documents, it was recommended for patients with mild asthma and infrequent symptoms (e.g., those with symptoms less than twice a month) should be treated only with SABA, a treatment that does not address at all the inflammatory basis of the disease [14]. Regular low dose ICS is highly effective in reducing symptoms and improving asthma control; however, adherence with ICS is poor, especially in patients with mild asthma. Importantly, a significant proportion of patients with good asthma control tend to use their controlled inhalers only when they are symptomatic [13].

While the need for continuous ICS-based controller treatment with as-needed reliever treatment is the mainstay of the management of moderate-to-severe asthma, the management of mild asthma has gone through a lot of controversies. Importantly, there is a clear need for refinement of reliever treatment, especially regarding the use of SABA in mild asthma. This fear for the overuse of SABA has been evaluated for more than 4 decades now, with reports of excess asthma deaths related to excessive use of SABA published in large case-control epidemiologic studies, that was later complemented by epidemiologic reports supporting a crucial role for regular ICS in the reduction in risk of asthma deaths [15,16]. Such evidence, in combination with data from randomized controlled trials in mild asthma, suggests that early intervention with regular use of low-dose ICS in mild persistent asthma decreased the risk of severe exacerbations and led to better asthma control [17]. The more recent National Review of Asthma Deaths in the UK showed that 9% of the patients with fatal asthma had mild disease treated by SABA alone, and overall, 39% of patients with fatal asthma had >12 SABA prescriptions, and 4% of them had >50 SABA prescriptions in the previous year, while at the same time 80% of the deceased patients with asthma and available information received <12 ICS prescriptions [18]. Additional real-life data suggest that there are clear unmet needs in patients with milder disease, as defined by the use of reliever-only therapy. In a population-based internet survey in Australia, 38% of a random sample of participants ≥16 years with current asthma were using the reliever-only medication, and one-quarter of them had made use of urgent asthma care in the previous year, suggesting that these patients need to be identified and managed more efficiently [19].

### 2.2. The Body of Evidence That Led the Change of Treatment Strategy in Mild Asthma

The harmful effects of SABA over-consumption in daily practice, in line with guidelines for mild asthma treatment, has led to the conduction of clinical trials that addressed the issue of the combined use of as-needed ICS/formoterol combination as a treatment strategy in mild asthma (Table 1).

SYGMA 1 [20] and SYGMA 2 [21] are both multicenter, phase III, randomized, double-blind, 52-week, placebo-controlled studies, involving patients 12 years or older with a clinical diagnosis of asthma for at least 6 months, who were assessed as needing GINA step 2 treatment. In the SYmbicort Given as-needed in Mild Asthma (SYGMA) 1 study, 3849 patients were randomly separated into three subgroups: (i) terbutaline group (twice-daily placebo plus terbutaline 0.5 mg used as needed), (ii) budesonide–formoterol group (twice-daily placebo plus budesonide–formoterol 200/6 μg as needed), (iii) maintenance group (twice-daily budesonide 200 μg maintenance plus terbutaline as needed) [20].

Primary efficacy results showed that budesonide–formoterol (BUD/FORM) as needed treatment was superior to terbutaline as needed, with 14% higher odds of having a week of well-controlled asthma; on the other hand, this treatment was inferior to budesonide maintenance therapy with regard to the percentage of electronically recorded weeks with well-controlled asthma per patient (34.4% vs. 44.4%). The BUD/FORM formulation as needed also resulted in a 64% lower rate of severe exacerbations compared to terbutaline as needed, and was equivalent to maintenance budesonide, but with an 83% lower median daily ICS dose; however, the Asthma Control Questionnaire (ACQ)-5 score was higher and the forced expiratory volume in 1 sec (FEV1) was lower in the BUD/FORM group compared to maintenance budesonide, albeit the differences were small and did not meet the minimum clinically important differences [20].

In the SYGMA 2 study, 4215 patients were divided into two groups: (i) those who received twice-daily placebo plus budesonide–formoterol 200/6 μg as needed and (ii) those with budesonide 200 μg maintenance therapy twice daily plus terbutaline 0.5 mg used as needed. In SYGMA 2, treatment with BUD/FORM as needed was non-inferior to the maintenance budesonide group for reducing severe asthma exacerbations, but with a 75% lower median daily ICS dose in the BUD/FORM group, with ACQ-5 and FEV1 changes similar to SYGMA 1 [21].

In addition to double-blind placebo-controlled clinical trials, open-label pragmatic studies were conducted to test in a real-life setting SABA versus ICS plus SABA versus BUD/FORM standard combination in mild asthmatics.

NOVEL START [10] was a 52-week, randomized, open-label, parallel-group, controlled trial, in which 675 patients aged between 17 and 85 participated. All participants were diagnosed with asthma and used a SABA as a single asthma treatment in the preceding 3 months. Study subjects were divided into three groups: (i) albuterol group: albuterol 100 μg twice used as needed for asthma symptoms, (ii) budesonide maintenance group: budesonide 200 μg twice daily plus as-needed albuterol 100 μg × 2 inhalations, and (iii) budesonide–formoterol group: budesonide–formoterol 200/6 μg used as needed. The primary outcome of this study was the annualized rate of asthma exacerbations. The annualized exacerbation rate in the budesonide–formoterol group was lower compared to the albuterol group (rate per patient per year 0.195 vs. 0.4, respectively, *p* < 0.001, i.e., 51% reduction) and did not differ significantly from the budesonide maintenance group (0.195 vs. 0.175, respectively, *p* = 0.65). Furthermore, the number of severe exacerbations was lower in the budesonide–formoterol group than the albuterol group (9 vs. 23, respectively, i.e., 60% reduction in severe exacerbation rate) and the budesonide maintenance group (9 vs. 21, 56% reduction in severe exacerbation rate) with >70% lower dose of glucocorticoids in the first group; however, maintenance budesonide demonstrated the greatest improvements in ACQ-5 scores [10].

PRACTICAL study [22] was also a 52-week, real-world, multicenter, open-label, independent, parallel-group, superiority, randomized controlled trial. All 890 participants aged 18–75 years, had a medical diagnosis of mild-to-moderate asthma and were treated with SABA as a reliever, with or without low-to-moderate maintenance doses of inhaled corticosteroids in the preceding 12 weeks. They were separated into two treatment arms, (i) those under budesonide–formoterol 200/6 μg × 1 inhalation as needed and (ii) those with budesonide 200 μg × 1 inhalation plus terbutaline 250 μg × 2 inhalations as needed. It was shown that the rate of severe exacerbations per patient per year was reduced with the as-needed use of budesonide–formoterol than in the other group (31% reduction). Additionally, the time until the first exacerbation was increased in the budesonide–formoterol group. The symptom control was approximately the same between the two groups [22].

### 2.3. New Recommendations from the Global Initiative for Asthma (GINA): The Revolution in Mild Asthma Treatment

On 12 April 2019, the date that the pocket guide of Global Initiative for Asthma (GINA) recommendations were released, a long-awaited breakthrough in asthma management was made [23]. This year’s report, released as a full document almost two months later, proposed the most fundamental changes in asthma therapy since the initial development of GINA recommendations almost three decades ago, especially regarding the approach to intermittent and mild asthma treatment.

According to the evidence and rationale presented in the above section, the 2019 document proposed that adults and adolescents with mild asthma should preferably be treated with ICS-containing regimens: as-needed low-dose ICS-formoterol in Step 1 and as needed low-dose ICS plus as-needed SABA or as-needed low-dose ICS-formoterol in Step 2.

**Figure 1:**
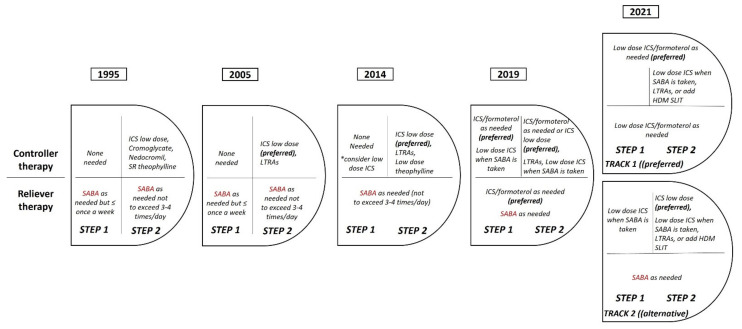
The evolution of SABA therapy in GINA guidelines for mild asthma over the last decades.

GINA Step 1 recommendations are for patients with symptoms less than twice a month, and no exacerbation risk factors, the less studied group of asthmatics. Low dose combination ICS-formoterol was taken as needed for relief of symptoms, and if needed before exercise is the Preferred Step 1 controller for adults and adolescents. It must be mentioned that, so far, all evidence for as-needed ICS-formoterol in mild asthma is with low dose budesonide–formoterol, but BDP-formoterol may also be suitable based on the experience of Steps 3–5 that are also used in an as-needed basis apart from standard treatment. Furthermore, GINA 2019, for the first time, recommended against SABA-only treatment of asthma in adults or adolescents (Figure 1) [24].

Accordingly, in Step 2, daily low dose ICS plus as-needed SABA in all age groups and as-needed low dose ICS-formoterol in adults and adolescents were the preferred controller options.

The withdrawal of the recommendation for on-demand short-acting β2 agonists (SABA) as monotherapy as the first step of asthma treatment evoked many positive comments, and experts’ opinion was rather enthusiastic on the implementation of this new therapeutical strategy in daily practice [24,25]. Most opinion leaders disagreed with the delay in the introduction of combined ICS/LABA use in mild asthma therapy as it was a concern among many asthma treating physicians that the use of SABA monotherapy may be associated with increased risk of exacerbations and death.

GINA 2021 document also recommends that all adults and adolescents with asthma should receive “as needed” or regular ICS-containing controller treatment, to reduce the risk of serious exacerbations. As stated, this is a population-level risk reduction strategy as already implemented with other drugs (e.g., statins and anti-hypertensives). Nevertheless, the current GINA treatment figure for the first time shows two ‘tracks’, based on evidence about outcomes with the two reliever choices across asthma severity: Track 1, with low dose ICS-formoterol as the reliever, is the preferred approach while Track 2, with SABA as the reliever, is an alternative approach when Track 1 is either not possible or not preferred by a patient that is well controlled on current controller medication without exacerbations. In Step 1 Track 2, ICS should be taken whenever SABA is used for relief. Moreover, good treatment adherence is mandatory for Track 2, while it is plausible that over-perception or under-perception of asthma symptoms remains a challenge in the ‘as needed” approach. Furthermore, treatment is more flexible as it may be stepped up or down within a track using the same reliever at each step, or switched between tracks, according to personalized needs and preferences [26].

Conclusively, from 2019 the as-needed or regular ICS-containing controller is incorporated into the patient’s personalized asthma management in all steps. A more precise definition of mild asthma that currently is based arbitrarily on the level of control with treatment may help to promote earlier recognition of mild asthma in daily practice as well as in clinical trials with better patient selection.

## 3. Towards the Minimization of Use of OCS in Severe Asthma

### 3.1. OCS in Severe Asthma: Effectiveness and Safety

The introduction of synthetic steroids in the middle of the 20th century changes the treatment perspectives of many diseases, including asthma. The efficacy of these drugs in the management of severe asthma has been documented in innovative studies in the early 1950s [27]. Following this, oral tablets of corticosteroids (OCSs) became the treatment of choice in asthma exacerbations and in severe forms of asthma for more than 20 years, being essentially the only choice of therapy other than bronchodilators. Inhaled corticosteroids (ICSs) have been used later in asthma treatment in the early 1970s, in the context of accumulating evidence that asthma is an inflammatory disease of the airways [28]. As the medical community began to recognize the side-effects associated with the prolonged use of steroids, OCS sparing was recognized as a primary outcome in these initial studies of ICS [29].

By 1960 all the toxic effects of chronic corticosteroid administration had been described and OCS-sparing strategies are utilized in nearly all diseases where OCSs are extensively used, not only for safety issues but also to improve outcomes [30,31]. There is abounded literature evidence on the relationship of long-term treatment of severe asthma with OCS and associated side-effects (risk of infections, gastrointestinal, neuropsychiatric, ocular, cardiovascular, metabolic, and bone-related complications), in both adults and children [6,32]. A clear dose–response relationship between maintenance OCS use and risk of related complications was demonstrated in both clinical and epidemiological studies [33,34]. In a recent metanalysis of 15 studies with more than 100,000 patients included, the risk of any complication increased in parallel with the dose of OCS, with pooled adjusted OR of 2.26 (95% CI 1.37–3.72), 2.94 (95% CI 2.62–3.29), and 3.35 (95% CI 2.94–3.82) for low dose (<6 mg), medium dose (5–12 mg), and high dose (>10 mg), respectively, compared with no OCS use [35]. Apart from continuous exposure to OCS, steroids bursts for asthma exacerbations have also been associated with a significant burden of adverse events in a dose-dependent way. Sullivan et al. reported that asthmatic subjects taking four or more OCS prescriptions within the year had 1.29 times the odds of experiencing a new AE within that year [36]. Moreover, in another study by Price and colleagues, a dose–response relationship for OCS exposure with most adverse outcomes have found to begin at cumulative exposures of 1.0 ≤ 2.5 g and for some outcomes (such as diabetes, obesity, and anxiety/depression) at cumulative exposures of only 0.5 ≤ 1 g (vs. > 0 ≤ 0.5 g reference), equivalent to four lifetime OCS courses [37].

A great deal of water has flowed under the bridge these last 50 years, new drugs and treatment strategies for asthma have been discovered, but OCS maintenance therapy still remains as an alternative option—even with a low grade of recommendation and reservations about their side effects—in the treatment algorithm of severe asthma [26]. The latest Global Initiative for Asthma (GINA) guidelines recommend the use of OCS for maintenance therapy only in patients with severe uncontrolled asthma despite treatment with all available controller drugs, including biologics if appropriate, and only as low-dosed and as short-term as possible [26]; however, real-world studies and data from severe asthma registries reveal that OCS use in asthma is much more widespread than one would expect.

The UK Severe Asthma Registry (UKSAR), one of the largest national severe asthma registries worldwide, recently published their updated data [38]. A total of 2225 adult patients with severe asthma were registered between November 2016 and February 2020 from 15 centers across the UK in this study. More than 50% of those patients (1142—51.7%) were on maintenance of oral corticosteroids (mOCS, with a median daily dose of 10 (IQR: 5, 15) mg prednisolone. This was observed even though a large proportion of them used additional controller therapy (53.5 on a long-acting antimuscarinic antagonist, 48.9% on a leukotriene receptor antagonist), and the majority (68.9%) were on therapy with a biologic. Similar data were reported last year from the International Severe Asthma Registry—ISAR [39]. The ISAR collected data on 4990 severe asthma patients between December 2014–December 2017 from the U.S., UK, South Korea, Italy, Australia, Singapore, and New Zealand. Further, in this registry, more than 50% of patients (51.1%) were on maintenance OCS, but the dose of this therapy was not reported in the manuscript. Similarly high rates of patients with severe asthma on OCS have been reported in the Italian (64%) [40], Belgian (24%) [41], and German registry (33.6%) [42].

Another significant issue in asthmatic patients is the frequent use of OCS bursts to treat asthma deterioration or exacerbations. Epidemiological data showed that a significant increase in the risk of severe side-effects could appear at levels as low as 0.5 g/year and became highly significant at levels >1 g/year [37,43]. In a recent study exploring expert consensus on oral corticosteroid tapering among 119 international experts, over 90% of them agreed or strongly agreed that the annual cumulative OCS dose should be monitored as a marker of asthma control and >75% of experts selected a threshold of 0.5 g or 1 g as the annual cumulative OCS dose indicative of poor control [8]. The magnitude of this problem in everyday clinical practice was highlighted in a study from Australia, focusing on the cumulative dispensing of doses associated with long-term toxicity (≥1000 mg prednisolone-equivalent) [44]. In this study, authors reported that cumulative exposure to OCS in Australia reaches levels associated with toxicity in one-quarter of patients with asthma using ICS, an extremely high and problematic number.

All the above data indicate an existing problem in the real-world management of severe asthmatics regarding OCS use. OCS should be considered a temporary option only, mainly for the treatment of asthma exacerbations—and to the least extent possible. Several options for stepping down from existing maintenance OCS treatment are recommended by GINA, including other controllers and especially biologic treatments as well as newer formulations and strategies of ICS/LABA regiments.

### 3.2. Steroid Sparing Effect of Biologics and New Concept of GINA Guidelines for OCS

Omalizumab (anti-IgE) has been extensively studied in clinical trials and real-world studies. We are lacking RCTs with OCS sparing effect of omalizumab. In real-life studies, omalizumab was found to reduce maintenance OCS but with a modest and slower rate—compared to the anti-IL-5/5R effect [45,46,47].

Many studies have proved the steroid-sparing effect of mAbs against IL-5/IL-5R.

In one of the first such studies, 20 asthmatics with corticosteroid-resistant eosinophilic asthma that were receiving a median dose of 10 mg of prednisone for a mean time of 9 years and high-dose ICS had >10% sputum eosinophils and received either mepolizumab or placebo at five monthly intravenous infusions. [48]. The study revealed a significant reduction in exacerbations along with a reduction in prednisone dose but no other clinically meaningful improvement in symptoms or lung function (FEV_1_).

The corticosteroid-sparing effect of mepolizumab was examined in the SIRIUS study. This study included 135 asthmatics with severe eosinophilic asthma receiving a mean dose of 10 mg of prednisone when entering the study. The study involved an “optimization” phase before the start of mepolizumab, during which a reduction in the dose of oral steroids was made until loss of control so as to achieve the lowest possible dose required for asthma control. The patients received either mepolizumab (100 mg SC) or placebo every four weeks for 20 weeks. The primary outcome was the percentage reduction in the oral corticosteroid dose, and the results of the study demonstrated that mepolizumab permitted the reduction in oral corticosteroid dose and also significantly reduced the rate of exacerbations and improved asthma control and quality of life [49].

A phase III study, ZONDA, showed the corticosteroid-sparing effect of benralizumab. All included patients were receiving oral corticosteroids for at least 6 months prior to entering the study. Similar to the SIRIUS study, this study had a run-in phase where the dose of oral steroids was attempted to be reduced to the minimum while maintaining asthma control before the start of benralizumab. Patients managed to reduce the dose of oral steroids significantly but also reduced the rate of exacerbations despite the reduction in OCS. The likelihood of reducing the dose was four times higher in the benralizumab group compared to the placebo group [50].

Dupilumab, an anti-IL-4 mAb was also assessed as an oral steroid-sparing agent. In a study including 210 patients, dupilumab reduced oral corticosteroid dose by 70% compared to 42% reduction in placebo (*p* < 0.001), and simultaneously reduced the rate of exacerbations by 59% compared to placebo [51].

A real-life study from Belgium included 116 asthmatics with severe eosinophilic asthma that were followed up at the asthma clinic for at least 18 months. A total of 25% of the patients were receiving maintenance oral corticosteroids and the mean dose was 8 mg prednisolone equivalent/day. Mepolizumab dramatically reduced the exacerbation rate by 85% and also allowed a reduction in the dose of maintenance oral corticosteroids by 50% over a period of 30 months [52].

Another real-life study from France included 146 patients with severe eosinophilic asthma who received at least one dose of mepolizumab. At baseline, 92.8% of the patients were using maintenance oral corticosteroids at a mean daily dose 20.6 ± 16.5 mg prednisolone equivalent. At the 12 and 24-month follow-up, only 41.1% and 34.7% of the patients were still using OCS, and those who still did, required lower doses. Approximately 33.0% and 62.5% of patients managed to reduce ≥50% the daily OCS dose at 6 and 12 months, respectively, with mepolizumab. Compared to the SIRIUS study, a similar reduction (≥50%) was observed in 54% of patients after 6 months of mepolizumab treatment; however, it should be taken into consideration that in contrast to randomized controlled trials (RCTs) where the reduction in OCS was performed according to a predefined protocol, in a real-life setting the reduction might be more gradual and slower and it is attempted under the guidance of the attending physician [53].

In another real-life study from the Netherlands with 76 severe eosinophilic asthmatics, 51.3% of patients at baseline were treated with systemic corticosteroids at a median daily dose (range) 10 mg prednisolone equivalent (3–50). At 6 and 12 months from baseline 24.3% and 15.4% of the patients treated with mepolizumab were receiving maintenance oral steroids. Compared to the SIRIUS study where 14% of the patients had a 100% reduction in OCS use after 20 weeks of mepolizumab treatment in the Dutch cohort 27% of the patients stopped OCS at 6 months and 35.9% after 12 months, indicating a greater benefit regarding steroid-sparing effect in the real-life setting [54].

Stepping down or stopping OCS is the first goal of every step in the management of difficult-to-treat asthma. If a severe asthmatic on maintenance OCS gains control after the modification of contributory factors or achievement of better adherence to treatment, the first thing to do is to step down the dose of OCS.

Initial asthma treatment may include a short course of OCS in a presentation of a patient with severely uncontrolled asthma or with an acute exacerbation.

Some patients do not take OCS regularly on a daily basis, but they take frequent short-term (5–7 days) courses due to frequent exacerbations. In that case, there is no need to taper the dose unless it has been given for more than 2 weeks [55].

In GINA step 5 treatment options, the addition of low dose OCS (<7.5 mg/day prednisone equivalent) is suggested for some patients, but this is Evidence D and the accompanying side effects are Evidence A [26]. The consideration of adding maintenance OCS should be taken into account after ensuring good inhaler technique, modification of other contributory factors, and after using other add-on treatments, including biologics—so, literally, it is the last option, Step 5, in cases where asthma remains uncontrolled. Prevention of osteoporosis should be considered in those asthmatics that are expected to take OCS for more than 3 months.

The goal remains the reduction in OCS and possible options include the use of a sputum-guided approach, the alternate-day OCS treatment, and the replacement of OCS with high-dose ICS. Still, the better and most valuable option is the recognition of a T2-high endotype and the use of a relevant biologic that will provide the opportunity to reduce or even stop OCS. As it was shown from the RCTs and real-world studies mentioned above, the reduction or even cessation of maintenance corticosteroids is a primary outcome of these studies (Table 2).

Patients on long-term OCS should be assessed for adrenal suppression in case of significantly reducing or stopping the treatment and should be advised for extra corticosteroids during injury or surgery for up to 6 months after cessation.

PONENTE is a phase IIIb study including 598 patients with severe eosinophilic asthma on maintenance OCS who were treated with benralizumab. The study implemented a faster steroid tapering schedule for prednisone dosages ≥7.5 mg than previous studies, and also an evaluation of adrenal insufficiency and an algorithm to taper OCS dosage when prednisone dosage was ≤5 mg/day. The two primary endpoints were whether patients achieve 100% reduction in daily OCS or achieve OCS dosage ≤5 mg/day, if adrenal insufficiency prevented further reduction [56]. The study revealed that 62.2% of patients stopped OCS, while 80.6% either eliminated OCS or reduced the dose to less than 5 mg/day if adrenal insufficiency prevented further reduction. Moreover, the reduction in OCS was irrespective of the baseline blood eosinophil count. Adrenal insufficiency was observed in 60% of patients reaching an OCS dose of 5 mg/day initially, but this percentage dropped to 38.5% after 2–3 months [57].

This study, as mentioned above, provided an algorithm for managing adrenal insufficiency in case this prevented the further reduction in OCS from the dose of 5 mg/day. The clinical utility of such an approach is enormous because, as it was shown in the Delphi study (an expert consensus on the tapering of oral corticosteroids for the treatment of asthma), it was impossible to reach a unified approach among experts in the assessment of adrenal insufficiency, thus warranting the need for further research in the management of it [8].

**Figure 2:**
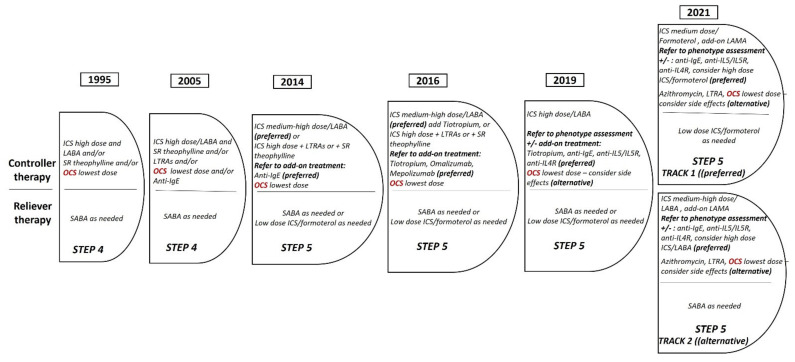
The evolution of OCS therapy in GINA guidelines for severe asthma over the last decades.

At the moment, OCS has moved underneath the dotted line for treatment options in GINA Step 5, indicating that the other treatment options are preferable (Figure 2) [26]. The future seems promising in such a way that OCS may be completely removed from the treatment regimen and very few asthmatics will require OCS, mainly during exacerbations. It should be kept in mind that in the UKSAR study, more than half of the severe asthmatics were on maintenance OCS despite biologic treatment in 69% of them. Still, since almost all available biologics present OCS-sparing effects, it remains a challenge to choose the proper mAb for each severe asthmatic. This requires extensive phenotyping, understanding of the underlying pathophysiologic mechanisms, and consideration of comorbidities.

## 4. Conclusions

There is no doubt that the recent changes in the GINA strategy will mark a new era of asthma management. This is paralleled and supported by the increasing acceptance and use of the “biologics”—monoclonal antibodies, blocking the key pathways involved in asthma pathophysiology—that appear to be safe and quite efficacious in cases that, only a few years ago, were desperate for any effective treatment. Currently, pricing is the major drawback of biologics and the only argument that may remain in favor of OCS. This is, of course, outdone in the long term. The proportion of patients with severe asthma that does not respond to biologics is small and is expected to decline with new agents coming in. There remains an unmet need both for patients with severe disease refractory, or partly responsive to biologics, as well as for ‘exacerbators’; this is of major significance when it comes to children.

On the other hand, the extension of compulsory anti-inflammatory treatment for the mild spectrum of the disease is reasonable and well-supported by evidence; however, it may be more difficult to achieve. Moreover, over-perception or under-perception of asthma symptoms may lead to over-use or under-use of asthma medication, and spirometry, when available, may assist in the evaluation of such poor-perceivers [58]. It is probable that this approach will descend the age barrier and be recommended to children as well; nevertheless, there are no data for this, while data on adolescents are scarce. Furthermore, although inhaled steroids have an excellent safety profile, potential side effects from cumulative long-term use cannot be excluded.

In all, bold changes in asthma management proposed by GINA, will hopefully improve further our rational approach to treatment and improve overall outcomes, for more patients. They will however take some time to be established. At the same time, we need to explore, most probably through real-life research and registries, the scope and pragmatic aspects of the new recommendations, while there is still space for additional interventions.

## Figures and Tables

**Table 1:** Studies comparing SABA vs. ICS/formoterol in mild asthma.

Study	Comparators	Patients	Duration	Results
O’ Byrne PM et al.	Budesonide–Formoterol as needed vs.	3849	52 weeks	Superior efficacy, reduced severe asthma exacerbations, and more weeks of well-controlled asthma in Budesonide–Formoterol group vs. Terbutaline group; with lower FEV_1_ and higher ACQ-5 score in Budesonide–Formoterol group vs. Budesonide group without important clinical significance
(SYGMA I study)	Terbutaline as needed vs.			
Randomized, double-blind, phase III study, multicenter	Budesonide maintenance plus Terbutaline as needed			
Bateman ED et al.	Budesonide–Formoterol as needed	4215	52 weeks	Non-inferior results in reducing severe asthma exacerbations between the two groups; higher ACQ-5 score and lower FEV_1_ values (without important clinical significance) in Budesonide–Formoterol group vs. Budesonide group
(SYGMA II study)	vs.			
Randomized, double-blind, phase III study, multicenter	Budesonide maintenance plus Terbutaline as needed			
Beasley R et al.	Budesonide–Formoterol as needed vs.	675	52 weeks	Lower asthma exacerbation rate in Budesonide–Formoterol group vs. Albuterol group; without important difference between Budesonide–Formoterol group and Budesonide group; ACQ-5 score better in Budesonide group
(NOVEL START study)	Albuterol (twice) as needed vs.			
Randomized, open-label, parallel group study multicenter	Budesonide maintenance plus Albuterol (twice) as needed			
Hardy et al.	Budesonide–Formoterol as needed	890	52 weeks	Reduction in annual asthma exacerbation rate in Budesonide–Formoterol group vs. Budesonide group with increased time to first exacerbation appearance and same symptoms control
(PRACTICAL study)	vs.			
Randomized, open-label, parallel group study, multicenter	Budesonide maintenance plus Terbutaline (twice) as needed			

**Table 2:** Studies on the OCS sparing effect of biologic therapies.

Study	Medication	Patients	Duration	Outcome
Bel et al.				Reduced oral corticosteroid dose (50%) and number of exacerbations (32%)
(SIRIUS study)				
Phase III	Mepolizumab	135	20 weeks	
Nair et al.				Reduced oral corticosteroid dose (50%) and number of exacerbations (55%)
(ZONDA study)				
Phase III	Benralizumab	220	28 weeks	
Rabe et al.				Reduced oral corticosteroid dose (50%), number of exacerbations (59%) and improved lung function (FEV1)
(VENTURE study)				
Phase III	Dupilumab	210	24 weeks	
Schleich F et al.				Reduced dose of maintenance oral corticosteroids (50%) Reduced exacerbation rate (85%)
Real life	Mepolizumab	116	>18 months	
Taile C et al.				Reduced or stopped (58%) OCS dose
Real life	Mepolizumab	146	24 months	
van Toor JJ et al.	Mepolizumab	76	12 months	Reduced or stopped (36%) OCS
Real life				
Menzies Gow A et al.	Benralizumab	598	52 weeks	Reduction (91%) or cessation (63%) of OCS Reduction in exacerbations
Open-label				
Single-arm				
